# Tissue- and Species-Specific Patterns of RNA metabolism in Post-Mortem Mammalian Retina and Retinal Pigment Epithelium

**DOI:** 10.1038/s41598-019-51379-3

**Published:** 2019-10-15

**Authors:** Les Kallestad, Seth Blackshaw, Ahmad M. Khalil, Krzysztof Palczewski

**Affiliations:** 1 Gavin Herbert Eye Institute and the Department of Ophthalmology, University of California-Irvine, Irvine, CA 92657 USA; 2 Department of Neuroscience, Johns Hopkins University, Baltimore, MD 21218 USA; 3 Department of Genetics and Genome Sciences, Case Western Reserve University, Cleveland, OH 44106 USA

**Keywords:** Genetics research, Molecular medicine

## Abstract

Accurate analysis of gene expression in human tissues using RNA sequencing is dependent on the quality of source material. One major source of variation in mRNA quality is post-mortem time. While it is known that individual transcripts show differential post-mortem stability, few studies have directly and comprehensively analyzed mRNA stability following death, and in particular the extent to which tissue- and species-specific factors influence post-mortem mRNA stability are poorly understood. This knowledge is particularly important for ocular tissues studies, where tissues obtained post-mortem are frequently used for research or therapeutic applications. To directly investigate this question, we profiled mRNA levels in both neuroretina and retinal pigment epithelium (RPE) from mouse and baboon over a series of post-mortem intervals. We found substantial changes in gene expression as early as 15 minutes in the mouse and as early as three hours in the baboon eye tissues. Importantly, our findings demonstrate both tissue- and species- specific patterns of RNA metabolism, by identifying a set of genes that are either rapidly degraded or very stable in both species and/or tissues. Taken together, the data from this study lay the foundation for understanding RNA regulation post-mortem and provide novel insights into RNA metabolism in the tissues of the mammalian eye.

## Introduction

**Figure 1:**
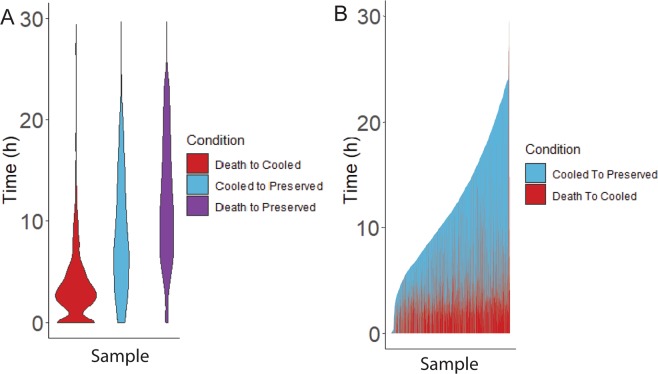
Ocular tissue donor collection stage times. Death to Cooled (Red), Cooled to Preserved (Blue), and Death to Preserved (Purple) collection stage times from 2019 Eye Bank Association of America data. (**A**) Violin plot displaying distribution of collection stage times. (**B**) Stacked bar graph displaying variance of collection stage times. n = 3567.

Several human ocular tissues are collected post-mortem and used for either transplants or biomedical research. Specifically, human eyes collected from deceased donors are often used for corneal transplants^1^. Donor eyes are also used for studies of the causes and progression of retinal diseases, such as age-related macular degeneration (AMD), which affects almost 11 million people in the US alone^2^. Gene expression changes are used as disease biomarkers and for the identification of molecular mechanisms controlling disease progression. It is critically important to distinguish such disease-related changes from those that result from differences in post-mortem interval (PMI), which is also referred to as death to preservation time. In the case of studies utilizing RNA from post-mortem samples PMI represents the time window from death to stabilization of RNA, either by cold storage or ideally by chemical RNA stabilization reagents such as RNAlater followed by immediate use or cold storage. Data obtained through personal communication with the Eye Back Association of America (EBAA) details the death-to-cooling and death-to-preservation times from 3,567 eye donors intended for corneal transplants. As death-to-cooling and PMI times of tissue collection vary (Fig. 1)^3,4^, along with the PMI of tissues used in research, a clear understanding of gene expression changes in the key cell types in the post-mortem eye is much needed and cannot be met by studies that use GTEx samples because GTEx does not collect retina or RPE and no other comprehensive post-mortem retina or RPE database exists^5^. It is also important to note that PMI times are reported in the literature utilizing post-mortem tissues, while death-to-cooling times are not reported in the literature, allowing for an unaccounted variable in these samples.

Gene expression is a tightly regulated process controlled by tissue-specific transcription factors, cofactors directing the binding of transcription factors, chromatin accessibility, and by various noncoding RNAs. This process results in the precise combination of transcribed mRNAs and protein production to maintain the physiological function of the cell and adaptation to external stimuli^6–8^, as steady state expression levels of individual genes are a sum of both the rate of de novo transcription and RNA degradation^9^.

Previous studies have been conducted on the degradation of RNA to determine the half-life of individual RNA species in many organisms and cell lines^10–14^. These studies provided insights into the stability of individual RNA species in living cells or organisms, identifying sequence-specific factors that correlate with RNA degradation^15,16^. These data provide evidence that RNA degradation is a tightly and actively regulated process, and that both *cis*- and *trans*-acting signals interact to enable different mRNAs to be degraded at specific rates^17^. Furthermore, some studies have characterized the mechanisms that control the degradation of RNA, including the identification of specific motifs that influence the process, giving us a greater understanding of this critical and complex process in living cells and organisms^16^.

While most studies have characterized the processes controlling RNA degradation in human cell lines in response to cellular cues and ATP-dependent processes, recent studies suggest that degradation of RNA post-mortem is also an active process^18–20^. Post-mortem RNA degradation in the absence of newly transcribed RNA results in the altered relative abundance of RNA levels and does not fully reflect the state of living tissues. In addition, it remains unclear how infiltration by immune cells, proximity to vascular tissue, or the complexity of different cell types within a tissue affects the transcriptome of neighboring cells in a living tissue. In this study we profiled retina and retinal pigment epithelium (RPE) from mice and baboon to illuminate the process of RNA degradation post-mortem and assess tissue- and species-specific differences in mRNA stability. It is important to note that primate retinas contain a macula, which is a cone rich area of the retina responsible for color vision and detailed perception, with the macula possessing altered function and cellular composition compared to the peripheral retina. While this study does not attempt to characterize the macula, further studies better characterizing macular RNA will aid in our understanding of primate post-mortem retinal tissues. Moreover, this investigation clearly demonstrates both cell type and species-specific patterns of RNA degradation post-mortem and illustrates the need for careful interpretation of gene expression in tissues obtained post-mortem compared to living tissues.

## Results

### Significant gene expression differences between retina and RPE/choroid

We initially characterized gene expression in the retina and RPE/choroid (enriched for RPE while clearly contaminated by choroid) immediately upon death from both mouse and baboon. These studies provide a baseline for gene expression in the retina and RPE/choroid in each species. Total RNA was isolated from both the retina and RPE/choroid from four C57BL/6 J mice (Mus musculus) and two baboons (Papio Anubis) immediately after death. Retinas from the same mouse were combined and treated as one replicate, as was the case for the RPE, whereas the retina and RPE from each baboon eye were treated as individual samples. Next generation RNA sequencing (RNA-Seq) was performed to quantify gene expression and analysis of RNA-Seq data identified mRNAs, long non-coding RNAs and pseudogenes that are expressed in each tissue type in each species as shown in Supplemental Tables 1, 2.

**Figure 2:**
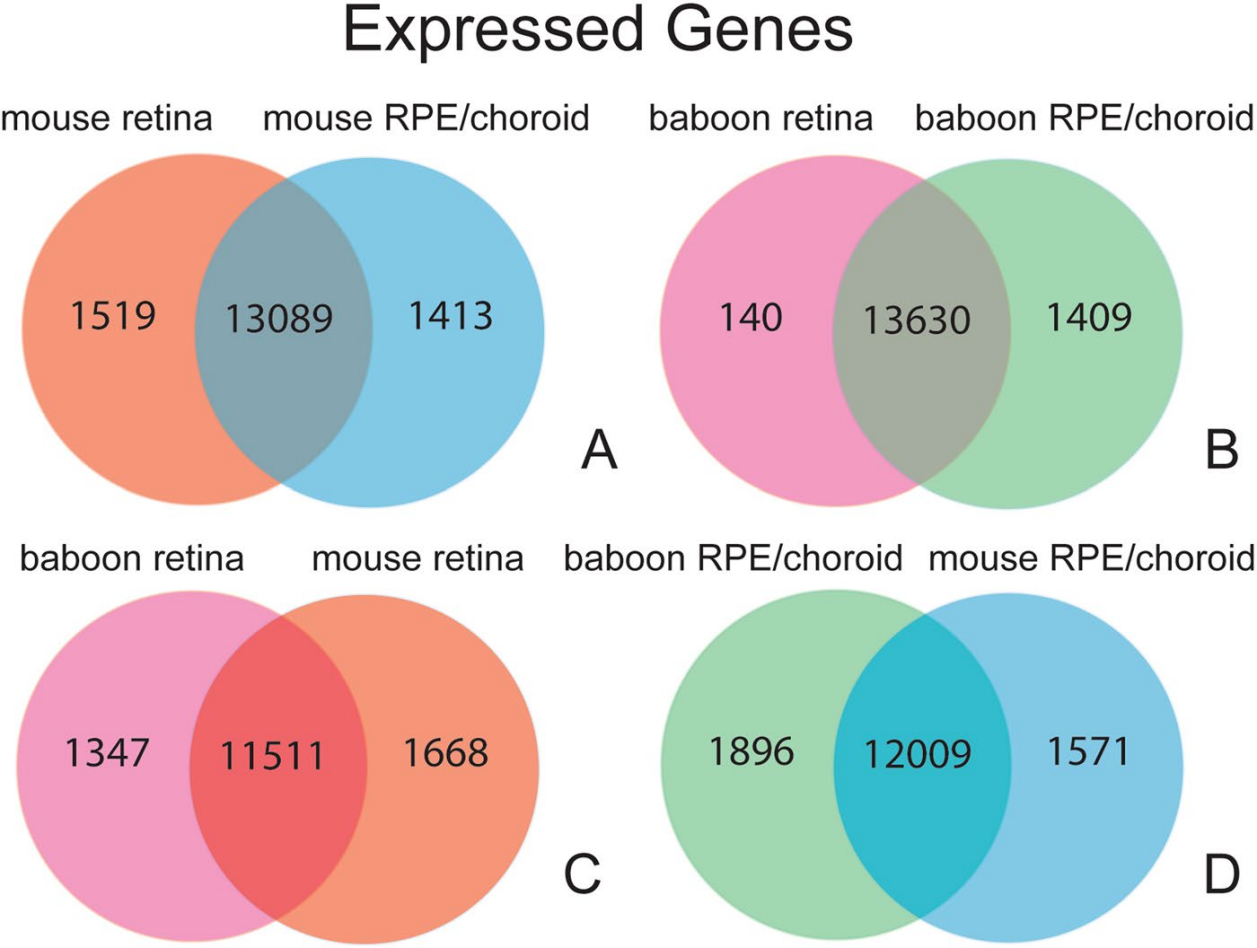
Gene expression analysis in baboon and mouse retina and RPE/choroid reveals tissue-specific gene expression. We analyzed gene expression in mouse and baboon retina and RPE/choroid by next generation RNA sequencing (RNA-seq) immediately after death. (**A**) While mouse retina and RPE/choroid share the expression of over 13,000 transcripts, each tissue shows unique expression of ~1,500 transcripts (Transcripts considered expressed at CPM >1). (**B**) Comparison of gene expression in baboon retina and RPE/choroid revealed a similar pattern to what we observed in the mouse (see (**A**). (**C**) Comparison of gene expression between mouse and baboon retina demonstrate that a unique set of genes are expressed in either species. (**D**) Similar to what we observed in retina, gene expression studies in mouse and baboon RPE/choroid demonstrate a significant overlap in gene expression but also over 1,500 transcripts that are uniquely expressed in either species.

To assess differences in gene expression between mouse retina and RPE/choroid, we first identified genes that are exclusively expressed, expression being defined by having a Counts Per Million (CPM), which is a standard normalized measurement of expression for RNA-Seq data, of 1 or greater, in either mouse retina or mouse RPE/choroid (i.e., these genes show less than 1 CPM expression in the other tissue) (Fig. 2A). We found ~1,500 genes that are unique to either the retina or RPE/choroid. We performed similar analysis for the baboon retina and RPE/choroid and observed a similar pattern (Fig. 2B). We also compared gene expression in the retina between mouse and baboon and found 1,347 genes uniquely expressed in baboon and 1,668 genes uniquely expressed in mouse (Fig. 2C). A similar analysis was done for RPE/choroid, with 1,896 genes uniquely expressed in baboon and 1,571 genes uniquely expressed in mouse (Fig. 2D). Lastly, differential gene expression analysis between the retina and RPE/choroid was performed which identified 14,484 genes that are differentially expressed (DE) in the mouse, and 4,728 differentially expressed genes in the baboon (q < 0.05) (Supplemental Fig. 1A,B).

The human retina contains 77.9–110 million rods and 4 million cones, 4.5% of human photoreceptors being cones^21^, while the mouse retina contains 6.4 million rods and 180 thousand cones, 2.8% of mouse photoreceptors being cones^22^. Despite primates having a macula, homogenized primate and mouse retinas contain a similar ratio of cell types, exempting cone enrichment. This allows for the first of its kind controlled cross-species analysis of RNA metabolism in a post-mortem setting for retina and RPE.

### Total RNA is stable post-mortem in retina and RPE/choroid

Many human tissue samples that are used for research are collected following substantial post-mortem intervals (PMIs). This includes human eye tissues. Previous studies have shown variable levels of RNA degradation in the human eye post-mortem including significant decrease and variability in RNA integrity numbers (RINs)^23–31^. Prior to RNA-Seq analysis, we therefore assessed RNA integrity (using RIN values) in post-mortem mouse and baboon retina and RPE/choroid. RNA samples were collected at the following post-mortem times from mice (0 h, 15 min, 30 min, 45 min, 60 min, 3 h, 6 h, 12 h, and 24 h) and from baboon (0 h, 3 h, 6 h, and 24 h). Mice were cervically dislocated and allowed to sit intact, with eyes remaining in the body, at room temperature until the required harvest time. Baboons were euthanized by administration of pentobarbital. After verification of no heartbeat and eye reflexes were fixed and dilated, baboon eyes were removed from the orbits as intact globes, placed in a sterile sealed container with sterile PBS, and stored at room temperature until the required harvest time.

Due to Case Western Reserve University’s protocols for handling mice and Southwest National Primate Research Center’s for handling baboons, the different organization’s species-specific requirements for euthanasia resulted in two distinct euthanasia methods used in this experiment. The authors acknowledge the unknown effect euthanasia methods may play in RNA metabolism and hope to address it comprehensively in a future report. To the author’s knowledge, there is no study directly addressing euthanasia and its effects on post-mortem RNA metabolism, thus it is beyond the scope of this manuscript.

The time points used in this study were chosen to reflect the PMI range of human samples currently used in research, to investigate RNA metabolism at early and late PMIs, and assess the impact of using samples from different PMIs, specifically those from diseased tissues that are potentially directly compared to control samples with different PMIs. Samples were stored at room temperature to control for the variable death to cooling time found in human samples and to analyze RNA degradation at room temperature in a tightly controlled experimental setting.

Mouse retina RIN values varied from 8.375 at the 0 h time point to 7.925 at the 24 h time point, and mouse RPE/choroid RIN valued varied from 9.125 at the 0 h time point to 7.675 at the 24 h time point (Supplemental Fig. 2). These values suggest that there is minimal post-mortem degradation of ribosomal RNAs (rRNAs) in mouse retina and RPE/choroid, which constitute over 90% of RNA in each cell. By comparison, baboon retina RIN values varied from 8.475 at the 0 h time point to 2.55 at the 24 h time point, while baboon RPE/choroid RIN values stayed stable from 7.66 at the 0 h time point to 6.35 at the 24 h time point. Thus, based on these limited samples, it appears that there are both tissue- and species-specific differences in ribosomal RNA metabolism post-mortem.

### Quantification of RNA levels in post-mortem retina and RPE/choroid tissues

To determine if the length of the post-mortem interval had an effect on the variation between biological replicates we calculated the biological coefficient of variation (BCV) in the retina and RPE/choroid for the mouse and baboon samples, which is the coefficient of variation with which the (unknown) true abundance of the gene varies between replicate RNA samples and it represents the coefficient of variation that would remain between biological replicates if sequencing depth could be increased indefinitely^32^. At earlier time points, the mouse retina maintained BCV values similar to the 0 h time point, with a trend of increasing values through the 12 h time point, and the 24 h time point displaying a higher BCV value. Mouse RPE/choroid levels had a higher initial BCV but stayed consistent up to 24 h (Supplemental Fig. 3A,B). Comparatively, baboon retina had an increased BCV relative to mouse retina displaying an increase over time, with a drop at 24 h. BCV values for baboon RPE/choroid were elevated relative to mouse RPE/choroid but maintained a similar level up to 24 h (Supplemental Fig. 3C,D).

**Figure 3:**
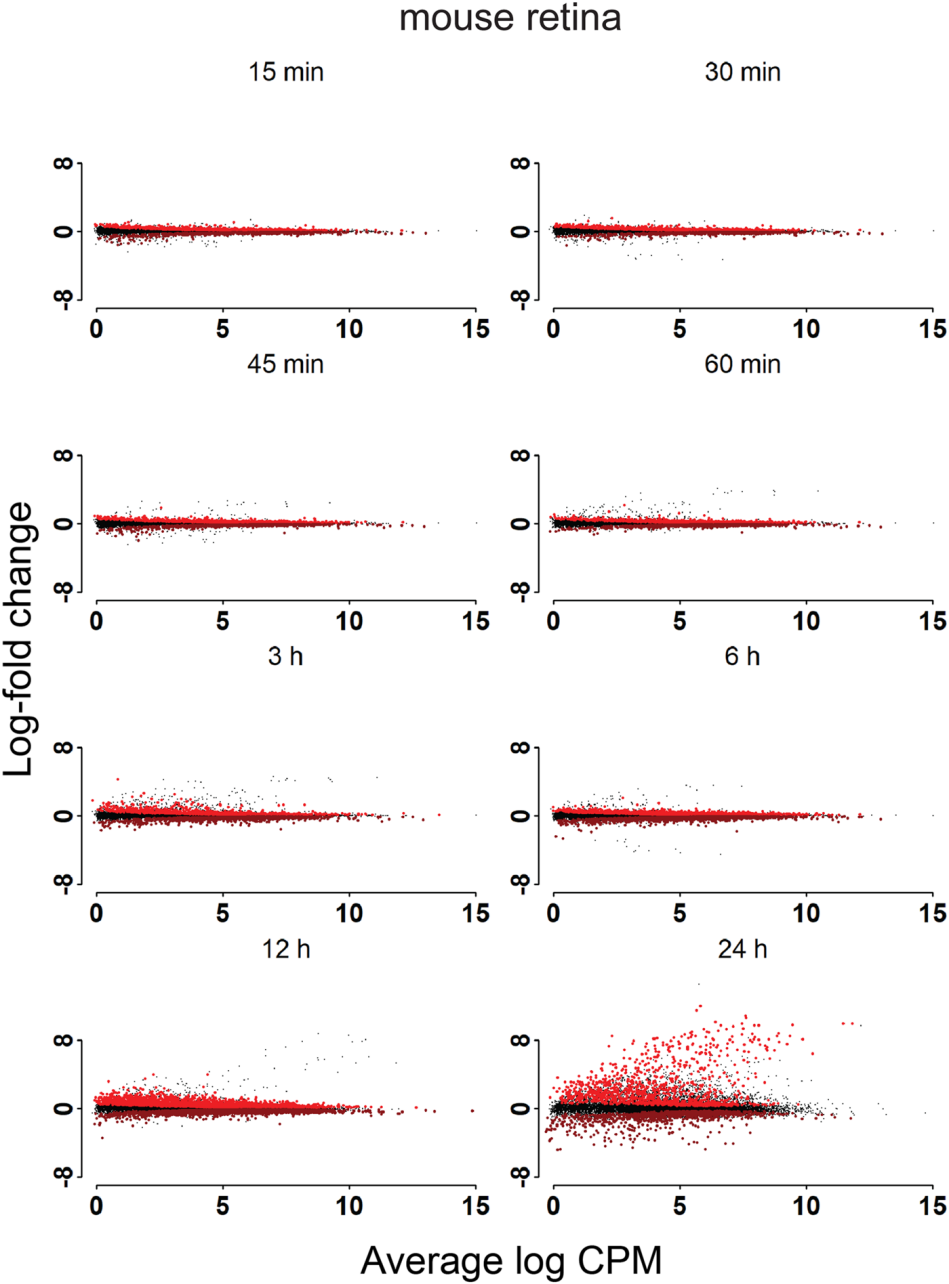
Post-mortem analysis of gene expression in mouse retina. Differentially expressed genes in mouse retina post-mortem compared to the zero-hour time point.

**Figure 4:**
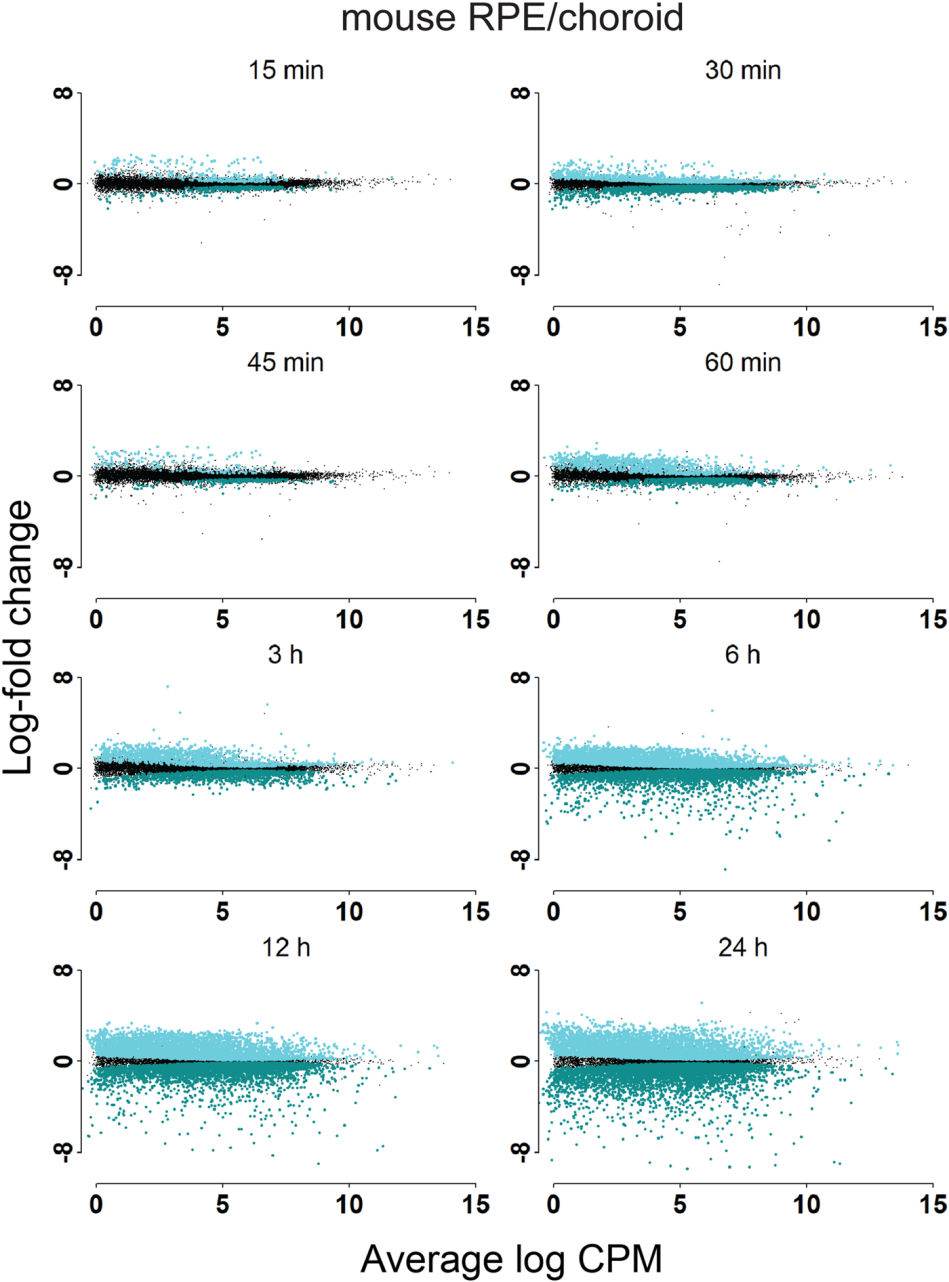
Post-mortem analysis of gene expression in mouse RPE/choroid. Differentially expressed genes in mouse RPE/choroid post-mortem compared to the zero-hour time point.

Distinct patterns of RNA degradation in RNA-seq data were analyzed by mapping reads to Ensembl 38.91 GTF files for mouse and Ensembl 3.0.92 GTF files for baboon, and glmQLFTest from edgeR, which does not use a fold change threshold, was used to identify DE based on statistical significance and a q value of <0.05. We generated MD plots for retina (Fig. 3) and RPE/choroid (Fig. 4) for all samples examined compared to the 0 h time point. Genes determined to be DE preserved (relatively upregulated) or DE degraded (relatively downregulated) are highlighted. In both mouse retina and RPE/choroid, we observed changes in RNA expression as early as 15 min post-mortem, with 1,021 genes down-regulated and 1,149 genes upregulated in the mouse retina and 342 genes down-regulated and 277 genes upregulated in the mouse RPE/choroid. As the PMI increased, the number of DE genes also increased, as indicated in Table 1. The largest number of DE genes was observed at 24 h post-mortem in the RPE/choroid. The number of DE genes in retina samples from mice increased over time, with a sharp drop at 24 h. This reduction of DE genes at 24 h is explained by the sharp increase in the BCV, resulting in less total DE genes despite an overall increase in log fold change (LFC) compared to 12 h.

**Table 1:** Number of mouse DE genes preserved or degraded relative to 0 h.

Time (h)	0.25	0.5	0.75	1	3	6	12	24
**Retina**								
Preserved	1149	1200	1295	1342	1262	2104	2905	1152
Degraded	1021	1026	1229	1189	1368	2351	2992	1047
Not DE	12950	12918	12598	12600	12506	10686	9340	13165
**RPE/choroid**								
Preserved	277	1513	169	915	1324	3544	4821	4852
Degraded	342	2228	204	691	1370	3869	4629	4767
Not DE	14493	11409	14788	13657	12500	7922	6017	5859

**Figure 5:**
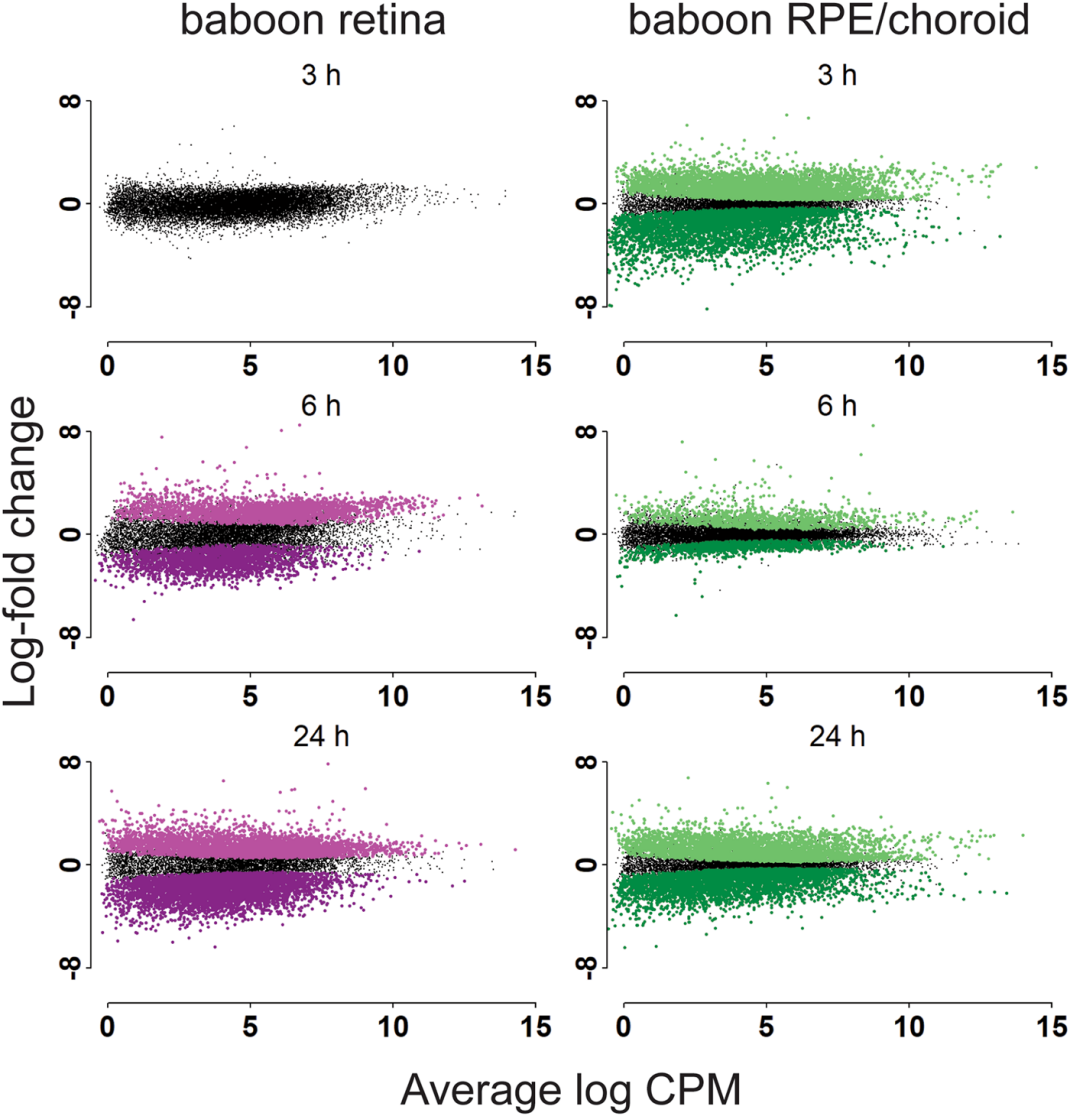
Post-mortem analysis of gene expression in baboon retina. Differentially expressed genes in baboon retina and RPE/choroid post-mortem compared to the zero-hour time point.

In our baboon studies, we had access to fewer time points including 0 h, 3 h, 6 h and 24 h post-mortem. In these studies, we observed little change in mRNA levels in the baboon retina at 3 h post-mortem, but substantial changes at 6 h and 24 h. Specifically, we observed 2,280 genes showing reduced and 2,689 genes showing increased expression at the 6 h time point, and 4,820 genes showing reduced and 4,243 genes showing increased expression at the 24 h time point. By contrast, we observed mRNA changes in the baboon RPE/choroid as early as 3 h post-mortem, with 4,258 genes showing reduced and 4,457 genes showing increased expression, with a similar number of preserved and degraded genes at the 24 h time point (Fig. 5 and Table 2).

**Table 2:** Number of baboon DE genes preserved or degraded relative to 0 h.

Time (h)	3	6	24
**Retina**			
Preserved	0	2689	4243
Degraded	0	2280	4820
Not DE	14690	9690	5777
**RPE/choroid**			
Preserved	4457	1150	4062
Degraded	4258	1112	4509
Not DE	6999	13383	7095

The number of DE genes between all individual time points for mouse and baboon in both retina and RPE/choroid were measured in order to determine the effect of using samples of varying time-points as replicates, as can be the case with rare samples of human diseased tissue. As expected, mice with neighboring PMI time points had the lowest number of DE genes, with multiple neighboring time points having no DE genes, and a consistent and steady increase in the number of DE genes as the PMI differences increased. In baboons, the trend in the number of DE genes was similar to what we observed in the mouse retina; however, the analysis in RPE/choroid showed less consistent trends. Additionally, any genes of interest could be checked against this data set to determine the potential masking or exaggerating effect of PMI differences between replicates or experimental conditions (Supplemental Fig. 4).

A subset of mouse retina and RPE candidate genes were selected (see Methods) to determine the ability to predict PMI of samples using a qRT-PCR assay. Four additional mice for each of the 0, 6 hr, 12 hr, 18 hr, and 24 hr PMI time points had retina and RPE samples collected and subjected to qRT-PCR for the 17 retina and 18 RPE candidate genes. (Supplemental Fig. 5, Supplemental Tables 3–5). Multiple linear regression models were generated for both retina and RPE to estimate the PMI. These data (Supplemental Table 6) suggest that RNA metabolism is tissue-specific and has a unique genetic signature correlated to the PMI of a sample.

### Analysis of preserved and degraded genes by species, tissue, and RNA class

**Figure 6:**
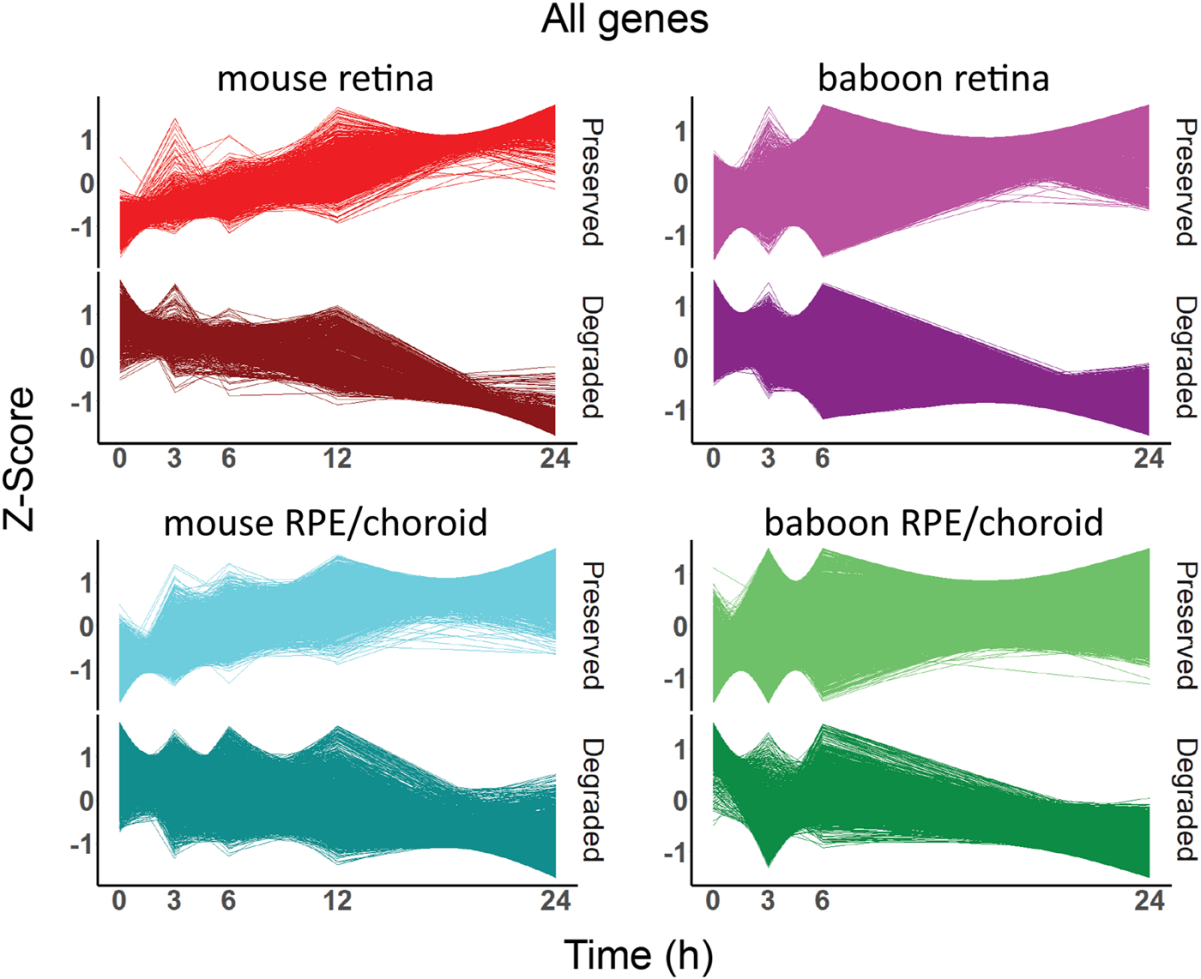
Z-Scores of preserved and degraded differentially expressed genes.

**Figure 7:**
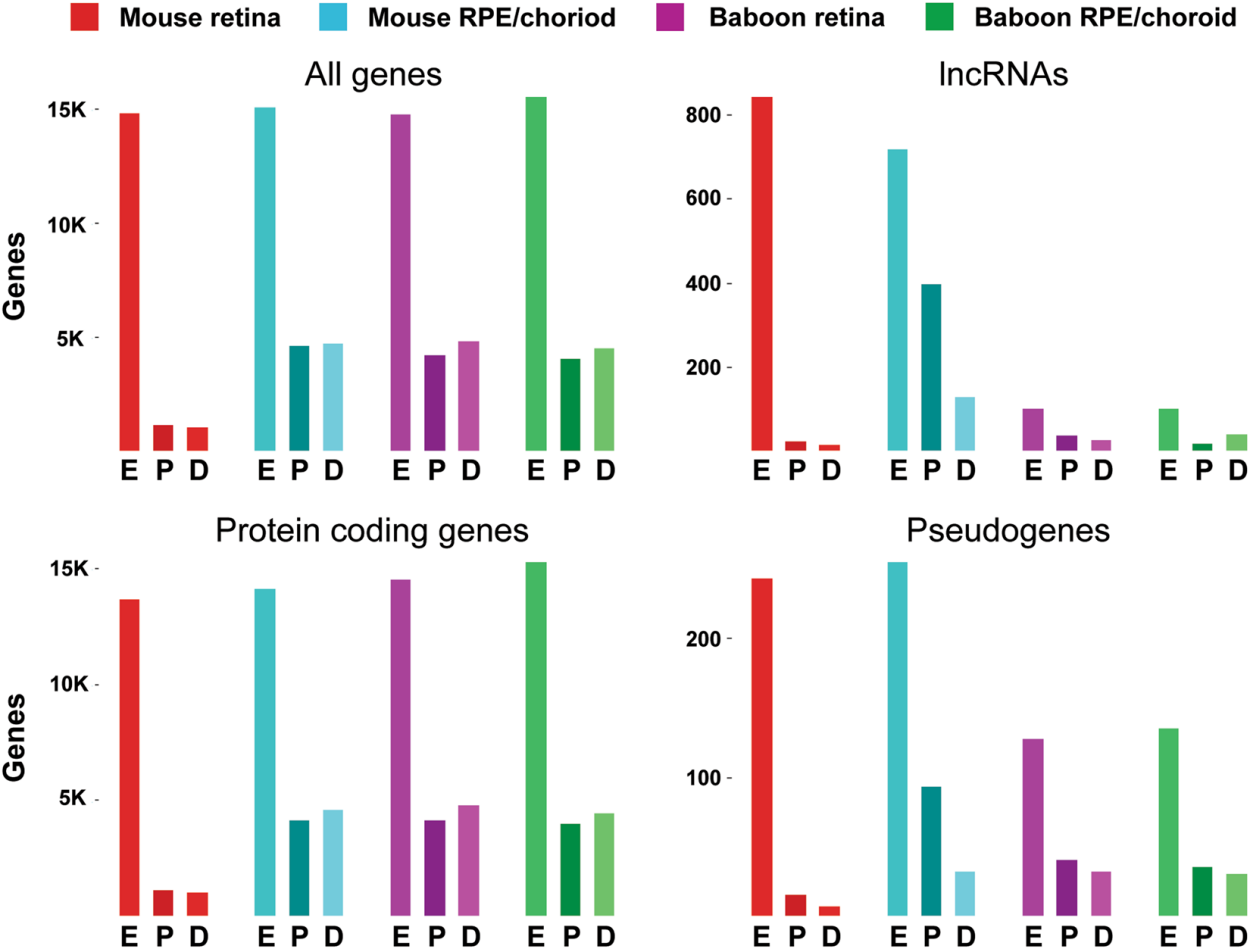
DE gene analysis of 24 PMI samples. Number of genes expressed (E) (CPM ≥1), preserved (P) (q ≤0.05) and degraded (D) (q ≤0.05) in mouse and baboon retina and RPE/choroid, presented by gene biotype.

Timeline plots of differentially expressed genes were generated for both mouse and baboon retina and RPE/choroid tissue, with preserved and degraded genes presented separately (Fig. 6). Similarly, timeline plots were created displaying only lncRNAs, pseudogenes, and mRNAs (Supplemental Figs 6–8). Additionally, the number of expressed genes, preserved genes, and degraded genes for each biotype was calculated (Supplemental Tables 1, 2). The results show an enrichment of lncRNAs preserved in the mouse RPE/choroid (55.4% of expressed genes) and a reduction of lncRNAs being degraded (17.9% of expressed genes). By contrast, lncRNAs in the mouse retina contributed only 2.6% of degraded RNAs and 1.8% of preserved RNAs of total expressed genes respectively. Additionally, increased percentages of pseudogenes in RPE/choroid are preserved relative to degraded for pseudogenes, providing evidence of tissue specific RNA regulation for these classes of RNA (Fig. 7).

Since it has been shown that lncRNAs have regulatory roles^33–35^, further analysis of lncRNAs in baboon versus mouse retina and RPE/choroid demonstrates both tissue- and species-specific differences in their PMI metabolism. While lncRNAs in the mouse RPE/choroid showed a global shift towards preservation, lncRNAs in baboon RPE/choroid showed a global shift towards degradation. In the mouse retina the overall number of lncRNA DE genes was reduced and showed a minor global shift towards preservation, while lncRNA in the baboon retina showed a global shift towards preservation. These data provide evidence for both tissue- and species-specific post-mortem regulation of lncRNA metabolism at a global level.

To further measure the effect of species- and tissue-type on post-mortem RNA metabolism, GO term enrichment was performed on preserved and degraded genes. GO term overlap between mouse and baboon was determined for both retina and RPE/choroid for the intervals of 0 h to 6 h PMI and 0 h to 24 h PMI (Supplemental Tables 7,8). Additionally, the overlap of preserved and degraded genes between the retina and RPE/choroid was determined for both mouse and baboon for both time comparisons (Tables 3, 4). All statically unaffected, preserved, and degraded genes were converted to their human homologs for the cross-species and cross-tissue comparisons to be conducted (see Methods). While retina and RPE/choroid are believed to have similar biological roles in both the mouse and baboon species, this analysis demonstrates species-specific RNA metabolism at the gene level, which contributes to the levels of functional proteins, evidenced by only a mild overlap of DE genes between species. Surprisingly, when performing intra-species analysis on the overlap of degraded and preserved genes between retina and RPE/choroid there is a 2.3 fold increase in the number of DE genes relative to an identical intra-tissue analysis, providing evidence that species-specific RNA metabolism plays a stronger role than tissue-specific RNA metabolism.

**Table 3:** Number of DE gene overlaps by species.

	0 h–6 h	0 h–6 h	0 h–6 h	0 h–24 h	0 h–24 h	0 h–24 h
	Overlap	Exclusively mouse	Exclusively baboon	Overlap	Exclusively mouse	Exclusively baboon
Retina preserved	120	1849	2227	322	772	3463
Retina degraded	305	1954	1917	485	512	4158
RPE/choroid preserved	94	3044	991	346	3737	3439
RPE/choriod degraded	109	3638	939	686	3827	3548

**Table 4:** Number of DE gene overlaps by tissue.

	0 h–6 h	0 h–6 h	0 h–6 h	0 h–24 h	0 h–24 h	0 h–24 h
	Overlap	Exclusively retina	Exclusively RPE	Overlap	Exclusively retina	Exclusively RPE
Mouse preserved	816	1153	2322	228	866	3855
Mouse degraded	798	1461	2949	520	477	3993
Baboon preserved	210	2137	875	1084	2701	2701
Baboon degraded	483	1739	565	1520	3123	2714

### Gene Ontology (GO) term analysis of preserved and degraded genes

These RNA-seq data sets of PMI require that preserved and degraded genes are independently analyzed for GO term enrichment, as opposed to traditional RNA-seq experiments which typically requires all DE to be analyzed together. In order to determine the set of genes preserved across both species and tissue types, all genes were first converted to their human homologs and genes common to all gene sets for either preserved or degraded genes were subjected to GO term enrichment using DAVID 6.8 (Supplemental Table 9). The disadvantage in converting genes to human homologs is the loss of genes without clear homologs and the ambiguity when a gene converts to multiple homologs. For these analyses genes without homologs were removed from the gene list, and genes with multiple homologs had the one with the lowest ensembl gene id number retained. 89% and 93% of mouse and baboon genes, respectively, mapped to human homologs, and were used for downstream inter- and intra-species analyses where noted. While the loss of partial information is undesirable, it allowed for cross-species GO term analysis and gene overlap analysis.

For degraded genes, terms relating to transcriptional regulation were enriched, i.e. transcription-DNA-templated, post-transcriptional regulation of gene expression, nucleus, transferase activity, DNA binding, and protein domain specific binding. In contrast, preserved genes reflected constitutively active cellular processes, i.e. intracellular signal transduction, cellular response to hormone stimulus, response to cAMP, response to mechanical stimulus, extracellular matrix, and membrane. These data demonstrate that RNA regulating different biological processes has distinct stability profiles.

## Discussion

Next generation RNA sequencing (RNA-Seq) technology has enabled the characterization of gene expression in numerous human tissues and cell types, which has in turn led to better classifications of cell types within complex tissues and their functions^36,37^. It has also enabled the identification of gene expression differences between healthy and diseased tissues^38^. However, one major challenge with gene expression studies of human tissues is that many investigations use tissues that were collected post-mortem^23–31,39^ and thus, how these findings reflect RNA expression levels of living tissues remains to be characterized^40^. A recent study using tissues obtained through GTEx (Genotype-Tissue Expression) analyzed the RNA expression of 41 human tissues post-mortem and provides access to tissue samples from donors with PMIs ranging from immediately after death up to 24 h post-mortem^41^. However, the authors concluded that human tissue sample collection PMI time and corresponding degradation level are dependent on the collection site. These differences in collection times are likely contributing to differences in gene expression among tissues analyzed, making it challenging to extrapolate these findings to healthy living tissues. Thus a clear understanding of how gene expression changes post-mortem in controlled experimental settings would aid in the design and interpretation of future experiments utilizing human post-mortem samples.

One primary purpose for studying diseased tissue by RNA-Seq analysis is to directly compare gene expression in altered phenotype samples to healthy samples while minimizing as many variables as possible. As human samples for some diseased tissues are difficult to obtain, controlling for the PMI is often not possible and samples from different PMIs are treated as biological replicates for the same condition in the literature^26,42^. Thus, there is an urgent need to determine the degree of RNA degradation post-mortem in a controlled setting. Since it is not feasible to do these studies in humans, we utilized two mammalian species–mouse and baboon–that are widely used in biomedical research of the eye.

Although they are neighboring tissues, retina and RPE/choroid have different biological roles in which they function to maintain healthy and viable ocular organs. In mice, while retina and RPE/choroid demonstrate ≈90% overlap of expressed genes, 14,484 of the 16,077 expressed genes showed differential expression between the two tissue types. In addition to the biological and genetic differences, retina and RPE/choroid have different tissue compositions. RPE, exempting potential choroidal contamination, is thought to be composed of a single cell type, whereas the retina is composed of five major neuronal cell types and up to 100 subtypes^43,44^, where each cell type possesses specific combinations of RNA levels and RNA degradation machinery^41,45^. The directed RNA degradation machinery of a single cell type, e.g. enriched RPE, with choroid contamination, would result in a uniform pattern, whereas multiple RNA degradation machinery combinations, i.e. retina cell types, would result in multiple effects of different trajectories and velocities, resulting in an averaging and masking of weaker signals. This could in part explain the reduction of DE genes in mouse retina compared to RPE/choroid.

Prior to examining post-mortem tissue samples, we characterized tissues harvested immediately following death in mouse and baboon retina and RPE/choroid tissues. This baseline in gene expression allowed comparisons between tissue types and species and enabled us to determine relative degradation levels of all RNAs present in the retina and RPE/choroid as compared to this 0 h time point. This body of research examined the effect of the PMI on the overall quality of the RNA through RIN analysis, its variability through BCV analysis, and the changes of expression in individual RNAs through RNA-Seq. Thus, to our knowledge, our current study represents the first comprehensive multi-species analysis of RNA metabolism in post-mortem mammalian eye utilizing next-generation sequencing technology.

When conducting biological experiments, variability between replicates can mask changes that might otherwise provide scientific insight. In this study we wanted to determine if the PMI effected the BCV, and what that effect might be. In well-controlled experiments, BCV is typically 0.4 for human data and 0.1 for data on genetically identical model organisms^46^. Our data matches the expected BCV at 0 h and shows that the PMI’s effect on BCV is minimal, except in the mouse retina at 24 h. One limitation in characterizing the BCV is the limited sample size of 4, whereas a larger sample size would better characterize the BCV for these species and tissues at the PMIs tested. We also examined the PMI’s influence on RIN values and surprisingly saw little to no effect on mouse retina and RPE/choroid up to the 24 h time point. Baboon data showed steady decreases in both tissue types tested with increased reduction in the retina. Interestingly, the baboon RIN data closely resembles what has been shown in the literature relating to degradation of RNA as the PMI increases^5,47^, while the mouse RIN data remained stable. The species-specific difference in retina RIN values can in part explain the increased number of differentially expressed genes seen in baboon retina at the 24 h PMI. While the RIN value primarily measures rRNA degradation levels, it is also used as a universally accepted indicator of mRNA degradation levels. In contrast to this, the RIN values for our mouse data would indicate the mRNAs are relatively undegraded and intact throughout the PMIs collected, while the sequenced data shows clear progression in the number of DE preserved and degraded genes, reflecting the altered mRNA abundance resulting from mRNA degradation. Additionally, our timeline analysis of DE genes shows clear, progressive preservation or degradation of individual genes. Understanding the reason for mouse RIN values remaining stable through the 24 h PMI while mRNA levels became altered due to gene specific global degradation would provide insight into overall RNA metabolism and the disadvantages and considerations required when using the mouse model for select experiments. Furthermore, this data provides evidence of RIN values incorrectly indicating mRNA quality in post-mortem conditions using mouse retina and RPE/choroid samples. The multiple regression models generated using the qRT-PCR data were better suited to quantify the level of RNA degradation in these samples. This body of research demonstrates tissue- and species-specific differences in RNA metabolism and details a tissue- and species-specific method superior to RIN values for determining RNA degradation in mouse retina and RPE samples.

While RIN values for mouse samples are not a good indicator for post-mortem mRNA degradation, the number of DE genes progresses as the PMI becomes larger, providing evidence for systemic mRNA change post-mortem even in the absence of reduced RIN values. Studies utilizing human retina vary in their death to preservation time, with studies utilizing samples with <6 h death to preservation^23–27^, studies utilizing samples with <24 h death to preservation^28,29^, and studies where the death to preservation time is not given^30,31^. Our mouse data shows that retina and RPE/choroid PMI samples from 3 to 6 hours can be treated as biological replicates while reducing the inter-sample variability. Our data suggest that samples with PMIs greater than 6 hours are still viable options for research on rare tissues, but consideration should be given to match PMIs both in biological replicates and to the control, and the increased BCV may reduce the statistical power of DE testing. Our dataset also provides quantification of changes in every gene between any of the PMIs collected, aiding the design and interpretation of experiments in which rare disease samples of varying PMIs must be used.

Mammalian genes maintain a high degree of homology and sequence conservation between species^48^, while enhancer and regulatory regions have rapidly adapted in a species-specific manner to create divergent function and morphology^49^. As lncRNAs are known to be involved in multiple aspects of genetic regulation, we further characterized the preservation and degradation of lncRNAs in post-mortem samples to gain insight into lncRNA metabolism^50^. One major species-specific difference observed in this data set is the number of expressed lncRNAs in mouse retina and RPE/choroid, 843 and 717 respectively, in contrast to baboon retina and RPE/choroid, 101 and 99, respectively, which is explained by lncRNAs currently being poorly annotated in the baboon species, with only 707 lncRNAs compared to 8897 lncRNAs in the mouse genome currently annotated.

Though intra-species analysis based on the number of lncRNAs is not possible due to differences in annotation levels, inter-species analysis of mouse lncRNA and pseudogenes indicates an enrichment in preserved transcripts in the RPE, but not the retina, relative to total expressed transcripts. As highly stable RNAs are typically enriched in housekeeping functions, and less stable RNAs are enriched in transcription and signaling factors, chromatin-modifying enzymes and genes with cell-cycle-specific functions^51^, our data show that mouse lncRNAs and pseudogenes have a similar ratio of preserved to degraded genes in a tissue-specific manner (Fig. 7)^51^, and supports the finding that pseudogenes can act in a regulatory manner mechanically equivalent to lncRNAs^52^.

While some mammalian genes are thought to maintain a high degree of homology^48^ the preservation and degradation of individual transcripts vary more by tissue species than by species tissue (Tables 3, 4). Similarly, GO term enrichment analysis also varies more by tissue species than by species tissue (Supplemental Tables 7, 8), indicating that transcript level species differences are not randomly selected but systematically programmed, with species level programming outweighing tissue level specification. While the mechanistic explanation for these species and tissue differences are beyond the scope of this manuscript, we believe our data to be the first to contrast species and tissue differences of RNA metabolism in a controlled and directed experimental setting. Better understanding the driving factors of species level global differences of RNA metabolism coupled with transcript level motifs will help elucidate steady state system dynamics and perturbations from this steady state which can lead to disease phenotypes.

## Materials and Methods

### Mice and baboon

All experiments were approved by the Institutional Animal Care and Use Committees at Case Western Reserve University (IACUC protocol No. 2014-0071) and University of California, Irvine (IACUC protocol No. AUP18–124), and were conducted in accordance with the Association for Research in Vision and Ophthalmology Statement for the Use of Animals in Ophthalmic and Visual Research. Male C57BL/6J mice were obtained from the Jackson Laboratory and handled at Case Western Reserve University. Baboon tissue samples were obtained from Southwest National Primate Research Center and processed at Case Western Reserve University. The baboons were 11-year-old females and had no known family relationship. Mice were delivered at 21 days of age and sacrificed at 30 days of age. Four mice were allocated to each PMI. Mice were cervically dislocated and allowed to sit at room temperature (RT) until the determined harvesting times of 0 h, 15 min, 30 min, 45 min, 60 min, 3 h 6 h, 12 h, or 24 h. An additional 4 male C57BL/6J mice of 30 days of age were cervically dislocated and had their orbits extracted upon death and stored at RT in sterile PBS in 1.5 microcentrifuge tubes until the 24 h PMI. Extraction of tissues from Baboon was performed by staff at the National Primate Research Center, and intact eye globes were collected at 0 h and stored at RT in sterile PBS in a sealed 50 mm conical tube until the required PMI was reached.

### Isolation of retina and RPE/choroid

Mouse and baboon retinas were extracted, and the RPE/choroid was isolated according to published protocols^53^. In brief, for mice 100 µl of RNAprotect (Qiagen, Hilden, Germany) and 100 µl of RNAlater (Qiagen, Hilden, Germany) were each placed in a 1.5 ml microcentrifuge tube. Under a dissecting microscope, a spring scissors was used to puncture the eye and remove the cornea, iris, and lens. The remaining eyecup had 4 incisions made every 90 degrees, resulting in a flat and open eye cup. The retina was then gently removed using a curved tweezers and placed in a 1.5 microcentrifuge tube containing RNAlater. The remaining eyecup was placed in a 1.5 microcentrifuge tube containing RNAprotect. The second mouse eye was processed identically and pooled with the first eye from the mouse. The tube containing RNAprotect and the 2 mouse eyecups was agitated by flicking with a finger after 10-minute RT incubation, then again after another 10-minute RT incubation. After the second agitation the eyecups were removed, with the dissociated RPE cells remaining in solution. Retinas and RPE samples in RNAlater and RNAprotect, respectively, were stored at 4° for up to one week. Baboon samples were processed as described for mice except each baboon eye was treated as an individual sample and the volumes of the RNA stabilization solutions was increased. Baboon retinas were placed in sterile 15 ml conical tubes and filled to the 15 ml line with RNAlater. Baboon eyecups were placed in sterile 50 ml conical tubes and filled to the 50 ml line with RNAprotect. Baboon retina and RPE samples were stored at 4° for up to one week.

### RNA isolation

Retina tissue samples were removed from the RNAlater solution and placed in a fresh 1.5 ml microcentrifuge tube. RPE samples were centrifuged for 5 minutes at 700 × g. After centrifugation the supernatant was discarded. All mouse RNA isolation was performed with the miRNAeasy micro kit with an optional DNase step, per the manufacturer’s protocol. Baboon retina RNA isolation was performed with the miRNAeasy mini kit, with an optional DNAse step, per the manufacturer’s instructions. Baboon RPE/choroid RNA isolation was performed with the miRNAeasy micro kit, with an optional DNAse step, as per the manufacturer’s instructions.

### RNA sequencing and data analysis

Total RNA was isolated as described above, quantified and analyzed on an Aligent 2100 Bioanalyzer to determine RIN prior to preparing libraries for sequencing. Libraries were made using the TruSeq stranded mRNA seq library kit (Illumina, San Diego, CA). RNA-seq was performed using an Illumina NextSeq 500 to generate 100 bp, paired-end, stranded sequences. Data is deposited as GEO accession number: GSE135415. Approximately 40 million reads per sample were generated. Alignment of sequences to the genome was completed using STAR version 2.5.3, ensembl GRCm38.p6 and Panu_3.0 were used for STAR mapping^54^, and read counts were generated using the featureCounts function of Rsubread^55^. Genes with 1≥ CPM in 4 or more replicates were considered expressed and used in all downstream analyses. Differential gene expression analysis was performed using edgeR^32,56^. Preserved and degraded DE gene timelines were created using a custom script and the R package ggplot2. Functional annotations were obtained using DAVID (Version 6.8)^57,58^.

To determine gene overlap between species homologous human genes were obtained by biomaRt using either baboon or mouse ensembl IDs as inputs. 89% and 93% of mouse and baboon genes, respectively, mapped to human homologs, and were used for downstream inter- and intra-species analyses where noted.

Genes which had a BCV of less than 0.075 for the 0, 3, 6, 12, and 24 hr time points had their Z score plotted from 0 to 24 hrs. The top genes that had visually consistent slopes, 9 positive and 9 negative, were selected for primer design and RT-qPCR (sup table X). The mean RT-qPCR cycle threshold (Ct) values for each biological replicate were determined and each gene had the replicate’s mean Ct value subtracted from its Ct value, providing an internal normalization for each biological replicate.

A custom R script was designed to perform multiple linear regression on every possible 3 gene combination of the 18. The models generated with a P value ≥ 0.05 were used to generate PMI estimates. The means of the PMI estimates were then determined and used as the model’s final PMI estimate.

## Supplementary information

SUPPLEMENTARY INFORMATION
