# Supplementary material for: Tissue- and Species-Specific Patterns of RNA metabolism in Post-Mortem Mammalian Retina and Retinal Pigment Epithelium
Source: Sci Rep. 2019 Oct 15;9:14821. doi: 10.1038/s41598-019-51379-3 (PMC6794289; doi:10.1038/s41598-019-51379-3)
Supplement: Supplementary file 1 — SUPPLEMENTARY INFORMATION [file 41598_2019_51379_MOESM1_ESM.pdf]

SUPPLEMENTARY INFORMATION

Tissue- and Species-Specific Patterns of RNA metabolism in Post-Mortem Mammalian Retina and Retinal Pigment Epithelium

Les Kallestad[1*], Seth Blackshaw[2], Ahmad M. Khalil[3], and Krzysztof Palczewski[1*]

[1]Gavin Herbert Eye Institute and the Department of Ophthalmology, University of California-Irvine, Irvine, CA 92657

[2]Department of Neuroscience, Johns Hopkins University, Baltimore, MD 21218

[3]Department of Genetics and Genome Sciences, Case Western Reserve University, Cleveland, OH 44106

Table S1. Number of genes expressed by tissue type and biotype.

**Mouse**	**All Transcripts**	**Protein Coding**	**lncRNA**	**Pseudogene**
retina	14608	13159	728	196
RPE/choroid	14502	13558	478	183
common	13089	12381	353	113
exclusively retina	1519	778	376	83
exclusively RPE/choroid	1413	1177	125	79
**Baboon**				
retina	13771	13530	66	110
RPE/choroid	15040	14730	82	128
common	13630	13399	60	110
exclusively retina	140	131	6	0
exclusively RPE/choroid	1409	1331	22	18

Table S2. Number of genes by biotype at 24 h PMI.

**Mouse retina**	**Expressed**	**Preserved**	**Degraded**
all genes	14750	1125	1033
protein coding	13664	1088	1011
pseudogene	243	15	7
lncRNA	843	22	15
**Mouse RPE/choroid**			
all genes	15041	4608	4709
protein coding	14069	4118	4549
pseudogene	255	93	32
lncRNA	717	397	128
**Baboon retina**			
all genes	14709	4177	4802
protein coding	14481	4102	4746
pseudogene	127	40	32
lncRNA	101	35	24
**Baboon RPE/choroid**			

all genes	15501	4036	4469
protein coding	15267	3983	4401
pseudogene	135	35	30
lncRNA	99	18	38

Table S3. Genes and primer sequences used in multiple linear regression modeling.

Gene	Primer Sequence
Pex19 F	GAAGACGACCGGGAACTGG
Pex19 R	CGAAGAGAGCATCTTTGGCAGTA
Arhgap12 F	TCAAAGCATCAAGATCCAGGTCA
Arhgap12 R	TGCCCAAGAAGACAACCAGT
Coq9 F	CTACATTGAGCACTGGCCCC
Coq9 R	TGTACCAGTTAAAGTCAGTGGAC
Got2 F	AAAAGCGGCCGGTTCGTC
Got2 R	GGCAGAAAGACATCTCGGCT
Usp30 F	ATGGCAGATCTCGTCCTTTGA
Usp30 R	CTGACTGCTGCTCCAGGGAAT
Trappc3 F	TGGCACGGAGAGCAAGAAAAT
Trappc3 R	TGTTGTAGCCCATTCTGTCCAGC
Srp14 F	GTGTCTGTTGAGAGCCACGG
Srp14 R	TCAGTAGATTTGAATAGGCCATCTG
Tbc1d20 F	CACCACCTCAGGGATTTCATGG
Tbc1d20 R	CAAAGATGGTCCCCACCTCAG
Ppp4r2 F	AGCTGCGTTTGTCCATCCTC
Ppp4r2 R	GGCCCGTTTATATTAGACCTGTCA
Dis3l2 F	AGGCTTGAAGAGAGGAACACT
Dis3l2 R	CCCGATCACCATCCGGAGAA
Fxr2 F	TTATTCTGTCAACCACAGAAGCCC
Fxr2 R	CTGCTGCCAGCTGCTTACTT
Chd8 F	AGCTAAACACCATCACTCCTG
Chd8 R	TTGGCGGTTTGAACGTCTCT
Brd7 F	ATTGGACACAGCAAAAGAAGCAG
Brd7 R	ACACCTCAGCCTCTTCGGAGT

Med23 F	ATAAGCAGACCTTGAACATTGC
Med23 R	AATGAGCTGGCTGGAAAGGT
Ppp2r2d F	GCGGAAGAACTGTCAACTGGG
Ppp2r2d R	TCAACGGTGGAGATGATGTCG
Psmc4 F	GTACAAGCAGATTGGCATCG
Psmc4 R	CCCACCACACGGATAAATGC
Uba2 F	CACTGGCTTCTCCCACATCG
Uba2 R	TCTTTGGCAACCTGAGCCTTT
Dctn3 F	GCTGGACAGCGCTTCTATCAA
Dctn3 R	AGCATTGTAGTCTTGTTATACCCTT
Rab3gap1 F	AGCCTCCAGATATAGATGCCCT
Rab3gap1 R	AAATGAGGCCACGTTGTTGC
Kpna6 F	AGAGCTTCTGATGCACAACGAT
Kpna6 R	GCAAGGGAGGGCTGAACAAT
Mkrn2 F	CCAAGCAGTTTGAAAACCCAA
Mkrn2 R	CTTCTTCCCCATTCCCTGTTTGA
Arhgap35 F	ACATCGAAGCCACAGGACTAA
Arhgap35 R	AAGTCTTTCTCTGCCAGGTCC
Btbd1 F	CGATCGCATCAGGTTCACAGTTA
Btbd1 R	TCCAGTATCATTCTGTCCCAAGG
Pias4 F	ATTGGGGTGAAGCATCCTGAG
Pias4 R	TTCACCAGTGGGCAGATGAGG
Got2 F	AAAAGCGGCCGGTTCGTC
Got2 R	GGCAGAAAGACATCTCGGCT
Rnf185 F	TCTTCTGTTGGCCGTGTTTAC
Rnf185 R	ACTTTGTCTCGGCTGATGCC
Pdrg1 F	TGCCTCACCCTAAGACGAAG
Pdrg1 R	CGGTTTTCCTTGGGCTTCAA
Kdm1a F	AAGCCAGGGATCGAGTAGGT

Kdm1a R	CTGACGACAGCCATGGGATT
Hnrnpd F	GGATGAAGGCCATTCAAACTCC
Hnrnpd R	GTCCCAGCTAAGGCCTCCTA
Pex5 F	GTCCCAAGAATTCATCGCCG
Pex5 R	TCATCGTACCATCGATCAGC
Trim41 F	ATAGTGTGGTGCCATTGGAGG
Trim41 R	TCTGGCTCTTCAGCTCTGTCAC
Psme4 F	AAGATGAACGAACTCAGGGCA
Psme4 R	ATTTTCCACTGGAGCGATCTTA
Atp5h F	GGGGGTCGGTGAAGTATCC
Atp5h R	TCCACCAGGGCTGTGTATTT
Zmynd11 F	GCTGTTGACGTCCCTACCAT
Zmynd11 R	CTGCTCACTGTCTGCTCCAT
Fam50a F	AGCAGCGTGAGCAAATGGA
Fam50a R	TTCAGAGTCACAAGACCCACAGTA

Table S4. Internally normalized qRT-PCR Ct values for mouse retina.

	Rab3gap1	Kpna6	Mkrn2	Arhgap35	Btbd1	Pias4	Got2	Rnf185	Pdrg1
Retina 0 h-1	-0.231	-0.13	0.921	-0.654	-1.266	1.903	-1.622	1.031	0.524
Retina 0 h-2	-0.211	0.032	0.814	-0.651	-1.433	1.959	-1.775	1.106	0.406
Retina 0 h-3	-0.149	0.058	1.017	-0.449	-1.357	1.905	-1.485	1.151	0.185
Retina 0 h-4	-0.086	-0.047	1.109	-0.573	-1.167	1.919	-1.562	1.061	0.375
Retina 6 h-1	-0.095	0.229	1.279	-0.602	-1.413	2.332	-1.588	1.234	0.134
Retina 6 h-2	-0.073	0.577	1.136	-0.658	-1.65	2.634	-1.592	1.226	0.27
Retina 6 h-3	0.017	0.457	1.127	-0.519	-1.342	2.276	-1.464	1.022	0.045
Retina 6 h-4	-0.105	0.108	1.312	-0.728	-1.377	2.081	-1.726	0.67	2.276
Retina 12 h-2	-0.752	0.561	1.814	-1.832	-2.307	1.454	-1.984	0.467	-0.205
Retina 12 h-3	-0.68	0.705	1.896	-1.915	-2.255	1.42	-2.012	0.4	-0.017
Retina 12 h-4	-0.731	0.676	1.876	-2.146	-2.185	1.219	-2.135	0.071	-0.236
Retina 18 h-1	0.005	0.25	1.68	-0.672	-1.052	2.198	-1.547	0.849	0.341
Retina 18 h-2	-0.048	0.355	1.691	-0.614	-0.989	2.032	-1.68	0.998	0.456
Retina 18 h-3	0.063	0.395	1.606	-0.64	-0.956	2.032	-1.731	0.722	0.23
Retina 18 h-4	0.146	0.217	1.764	-0.695	-0.917	1.93	-1.768	0.565	0.444
Retina 24 h-1	-0.306	0.245	0.95	-0.781	-1.133	1.836	-1.545	1.028	0.699
Retina 24 h-2	-0.245	0.155	1.378	-0.847	-0.998	1.794	-1.696	1.093	0.682
Retina 24 h-3	-0.35	0.133	1.356	-1.072	-1.046	1.952	-1.663	1.239	0.792
Retina 24 h-4	-0.406	-0.074	0.954	-1.18	-1.088	1.42	-1.958	0.762	0.781

	Kdm1a	Hnrnpd	Pex5	Trim41	Psme4	Atp5h	Zmynd11	Fam50a
Retina 0 h-1	-0.049	-0.44	1.512	1.564	0.589	-3.839	-0.769	0.963
Retina 0 h-2	-0.073	-0.349	1.591	1.592	0.682	-3.854	-0.812	0.977
Retina 0 h-3	-0.194	-0.57	1.464	1.525	0.512	-3.97	-0.597	0.955
Retina 0 h-4	-0.11	-1.025	1.421	1.439	0.416	-3.695	-0.558	1.087
Retina 6 h-1	-0.392	-0.489	1.675	1.739	0.179	-4.049	-0.884	0.713
Retina 6 h-2	-0.471	-0.643	1.858	1.492	0.061	-3.889	-0.979	0.7
Retina 6 h-3	-0.268	-0.491	1.906	1.603	0.087	-4.13	-0.962	0.637
Retina 6 h-4	-0.527	-0.778	1.315	1.141	0.405	-3.712	-1.147	0.793
Retina 12 h-2	-0.155	-1.451	0.276	1.027	1.922	-3.144	-1.52	5.822
Retina 12 h-3	-0.297	-1.415	0.236	0.883	1.932	-3.142	-1.534	5.781
Retina 12 h-4	-0.181	-1.37	-0.082	0.532	2.522	-3.091	-1.608	6.865
Retina 18 h-1	-0.408	-0.723	1.409	1.263	0.175	-3.677	-0.994	0.903
Retina 18 h-2	-0.443	-0.782	1.349	1.195	0.243	-3.709	-0.993	0.939
Retina 18 h-3	-0.212	-0.602	1.38	1.387	0.282	-3.996	-0.909	0.948
Retina 18 h-4	-0.418	-0.723	1.303	1.175	0.43	-3.801	-0.848	1.194
Retina 24 h-1	-0.096	-0.43	1.49	1.434	0.07	-3.361	-0.803	0.695
Retina 24 h-2	-0.255	-0.61	1.025	1.342	0.235	-3.492	-0.566	1.01
Retina 24 h-3	-0.272	-0.535	1.07	1.309	0.133	-3.238	-0.814	1.008
Retina 24 h-4	-0.407	-0.622	1.022	0.904	0.401	-3.294	-0.896	0.82

Table S5. Internally normalized qRT-PCR Ct values for mouse RPE.

	Pex19	Arhgap12	Coq9	Got2	Usp30	Trappc3	Srp14	Tbc1d20	Ppp4r2
RPE 0 h-1	-1.370	1.348	-1.122	-2.281	1.010	-0.144	-2.246	0.308	-0.497
RPE 0 h-3	-1.432	1.700	-0.815	-2.467	0.830	-0.278	-2.045	0.279	-0.350
RPE 0 h-4	-1.504	1.874	-0.911	-2.614	0.944	-0.211	-2.068	0.229	-0.606
RPE 6 h-1	-1.371	1.846	-0.179	-1.923	1.617	0.213	-2.405	0.495	-0.633
RPE 6 h-2	-1.096	2.184	0.101	-2.056	1.579	0.361	-2.133	0.560	-0.634
RPE 6 h-3	-1.175	2.136	0.031	-2.012	1.525	0.400	-2.146	0.440	-0.463
RPE 6 h-4	-1.340	1.986	-0.227	-2.146	1.480	0.324	-2.246	0.390	-0.631
RPE 12 h-2	-1.187	2.015	0.074	-1.789	1.454	0.306	-1.790	0.313	-0.766
RPE 12 h-3	-1.280	2.037	-0.109	-1.933	1.371	0.243	-1.802	0.162	-0.681
RPE 12 h-4	-1.289	1.750	-0.016	-1.474	1.838	0.299	-1.955	0.573	-0.905
RPE 18 h-1	-1.070	2.250	0.084	-1.911	1.550	0.581	-2.330	0.603	-0.482
RPE 18 h-2	-1.041	2.282	0.014	-1.917	1.619	0.235	-1.880	0.630	-0.627
RPE 18 h-3	-1.076	2.234	0.184	-1.938	1.611	0.504	-1.999	0.495	-0.535
RPE 18 h-4	-1.179	2.423	0.339	-2.116	1.680	0.706	-1.682	0.538	-0.556
RPE 24 h-1	-0.741	2.329	-0.223	-1.933	1.664	0.306	-2.030	0.754	-0.606
RPE 24 h-2	-0.889	2.583	0.174	-2.165	1.669	0.063	-2.434	0.878	-0.066
RPE 24 h-3	-0.869	2.763	-0.029	-2.171	1.761	0.474	-2.184	0.822	-0.131

	Dis3l2	Fxr2	Chd8	Brd7	Med23	Ppp2r2d	Psmc4	Uba2	Dctn3
RPE 0 h-1	0.688	0.090	-0.632	-0.731	1.836	7.727	-2.012	-0.008	-1.955
RPE 0 h-3	0.785	-0.274	-0.609	-0.427	1.750	6.766	-1.814	0.114	-1.714
RPE 0 h-4	0.929	-0.168	-0.517	-0.379	1.446	7.205	-1.838	0.039	-1.857
RPE 6 h-1	0.392	0.114	-0.719	-1.046	1.610	5.880	-1.784	-0.177	-1.932
RPE 6 h-2	0.220	-0.063	-0.770	-0.839	1.323	4.997	-1.723	-0.212	-1.798
RPE 6 h-3	0.230	-0.087	-0.786	-0.826	1.229	5.134	-1.853	-0.074	-1.703
RPE 6 h-4	0.339	-0.006	-0.674	-0.736	1.519	5.508	-1.643	-0.238	-1.661
RPE 12 h-2	0.099	-0.031	-0.845	-0.763	1.326	5.075	-1.509	-0.404	-1.579
RPE 12 h-3	-0.018	-0.012	-0.765	-0.669	1.486	5.331	-1.453	-0.274	-1.633
RPE 12 h-4	-0.021	-0.011	-0.920	-0.864	1.589	5.370	-1.701	-0.623	-1.641
RPE 18 h-1	0.123	-0.144	-0.808	-0.897	1.254	5.098	-1.753	-0.333	-1.814
RPE 18 h-2	-0.016	-0.137	-0.820	-0.836	1.333	5.045	-1.764	-0.247	-1.875
RPE 18 h-3	0.056	-0.148	-0.902	-0.885	1.272	5.119	-1.789	-0.367	-1.835
RPE 18 h-4	-0.236	-0.333	-1.000	-0.704	0.941	4.688	-1.529	-0.366	-1.616
RPE 24 h-1	0.332	-0.313	-0.689	-0.987	1.018	5.264	-2.020	-0.084	-2.041
RPE 24 h-2	0.180	-0.332	-0.673	-0.884	1.193	5.581	-2.224	-0.142	-2.515
RPE 24 h-3	0.075	-0.104	-0.609	-0.888	1.119	4.503	-2.059	-0.151	-2.323

Table S6. PMI estimates from multiple linear regression modeling.

Retina	0 h	6 h	12 h	18 h	24 h
replicate 1	1.406	-0.120	2.464	11.293	9.373
replicate 2	8.566	4.510	11.652	19.738	21.435
replicate 3	-3.796	-5.155	21.894	16.943	22.080
replicate 4	-6.075	24.656		23.967	43.128
mean	0.025	5.973	12.003	17.985	24.004
StDev	6.498	13.066	9.720	5.314	14.025

RPE	0 h	6 h	12 h	18 h	24 h
replicate 1	-4.091	-2.060	12.853	11.669	21.898
replicate 2	3.381	11.819	12.708	20.617	23.244
replicate 3	0.744	10.471	10.249	16.093	26.787
replicate 4		3.865		23.983	
mean	0.011	6.024	11.937	18.091	23.976
StDev	3.790	6.413	1.464	5.364	2.525

Table S7. Number of GO term overlaps by species.

	**0 h - 6 h Biological Process**	**0 h - 6 h Cellular Component**	**0 h - 6 h Molecular Function**	**0 h - 24 h Biological Process**	**0 h - 24 h Cellular Component**	**0 h - 24 h Molecular Function**
**Retina preserved**	97	36	18	133	25	21
**Retina degraded**	153	34	16	148	24	31
**RPE/choroid preserved**	27	10	24	60	20	11
**RPE/choroid degraded**	46	12	13	143	5	39

Table S8. Number of GO term overlaps by tissue.

	0 h - 6 h Biological Process	0 h - 6 h Cellular Component	0 h - 6 h Molecular Function	0 h - 24 h Biological Process	0 h - 24 h Cellular Component	0 h - 24 h Molecular Function
**Mouse preserved**	210	68	68	57	20	20
**Mouse degraded**	232	48	65	87	1	16
**Baboon preserved**	241	53	33	356	110	61
**Baboon degraded**	257	84	49	313	90	87

Table S9. GO term enrichment on cross-tissue and -species preserved and degraded genes

**Degraded**	**Biological process**	**Fold Enrichment**
GO:0007628	adult walking behavior	18.3430127
GO:0006486	protein glycosylation	6.953560372
GO:0006351	transcription, DNA-templated	1.777601899
GO:0035108	limb morphogenesis	44.32894737
GO:0033962	cytoplasmic mRNA processing body assembly	29.55263158
GO:0089711	L-glutamate transmembrane transport	27.27935223
GO:0010608	posttranscriptional regulation of gene expression	25.33082707
GO:0006488	dolichol-linked oligosaccharide biosynthetic process	23.64210526
GO:0042073	intraciliary transport	22.16447368
GO:0045776	negative regulation of blood pressure	19.70175439
**Degraded**	**Cellular component**	**Fold Enrichment**
GO:0005634	nucleus	1.298122353
GO:0032587	ruffle membrane	6.744484431
**Degraded**	**Molecular Function**	**Fold Enrichment**
GO:0016765	transferase activity, transferring alkyl or aryl (other than methyl) groups	115.4786325
GO:0004583	dolichyl-phosphate-glucose-glycolipid alpha-glucosyltransferase activity	86.60897436
GO:0046872	metal ion binding	1.734106272
GO:0003677	DNA binding	1.79235128
GO:0005313	L-glutamate transmembrane transporter activity	31.49417249
GO:0004843	thiol-dependent ubiquitin-specific protease activity	7.118545838
GO:0019904	protein domain specific binding	3.87079215
**Preserved**	**Biological process**	**Fold Enrichment**
GO:0035556	intracellular signal transduction	7.924728021
GO:0045944	positive regulation of transcription from RNA polymerase II promoter	4.421840137
GO:0060716	labyrinthine layer blood vessel development	74.65927978
GO:0000122	negative regulation of transcription from RNA polymerase II promoter	4.74423517
GO:0045597	positive regulation of cell differentiation	42.98564593
GO:0032870	cellular response to hormone stimulus	39.40350877
GO:0051591	response to cAMP	36.37246964
GO:0071277	cellular response to calcium ion	30.8375286
GO:0009612	response to mechanical stimulus	27.27935223
GO:0001503	ossification	23.25452977
GO:0006357	regulation of transcription from RNA polymerase II promoter	5.797791481
**Preserved**	**Cellular component**	**Fold Enrichment**

GO:0031012	extracellular matrix	12.39689387
GO:0016020	membrane	2.266365214
**Preserved**	**Molecular Function**	**Fold Enrichment**
GO:0000982	transcription factor activity, RNA polymerase II core promoter proximal region sequence-specific binding	75.06111111
GO:0000978	RNA polymerase II core promoter proximal region sequence-specific DNA binding	8.562103929

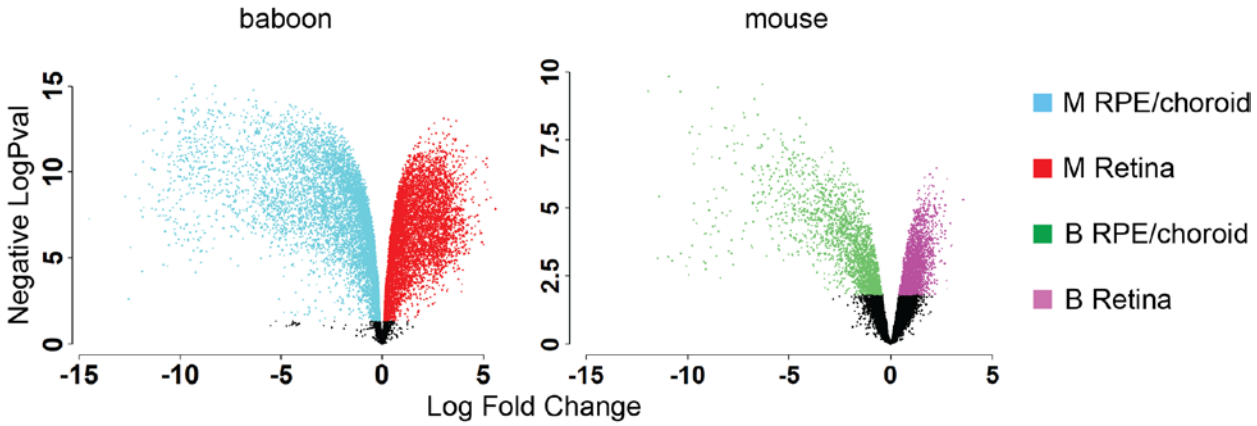

Figure S1. Volcano plots of retina vs RPE/choroid for M = mouse and B = baboon. Black dots represent genes not statistically differentially expressed.

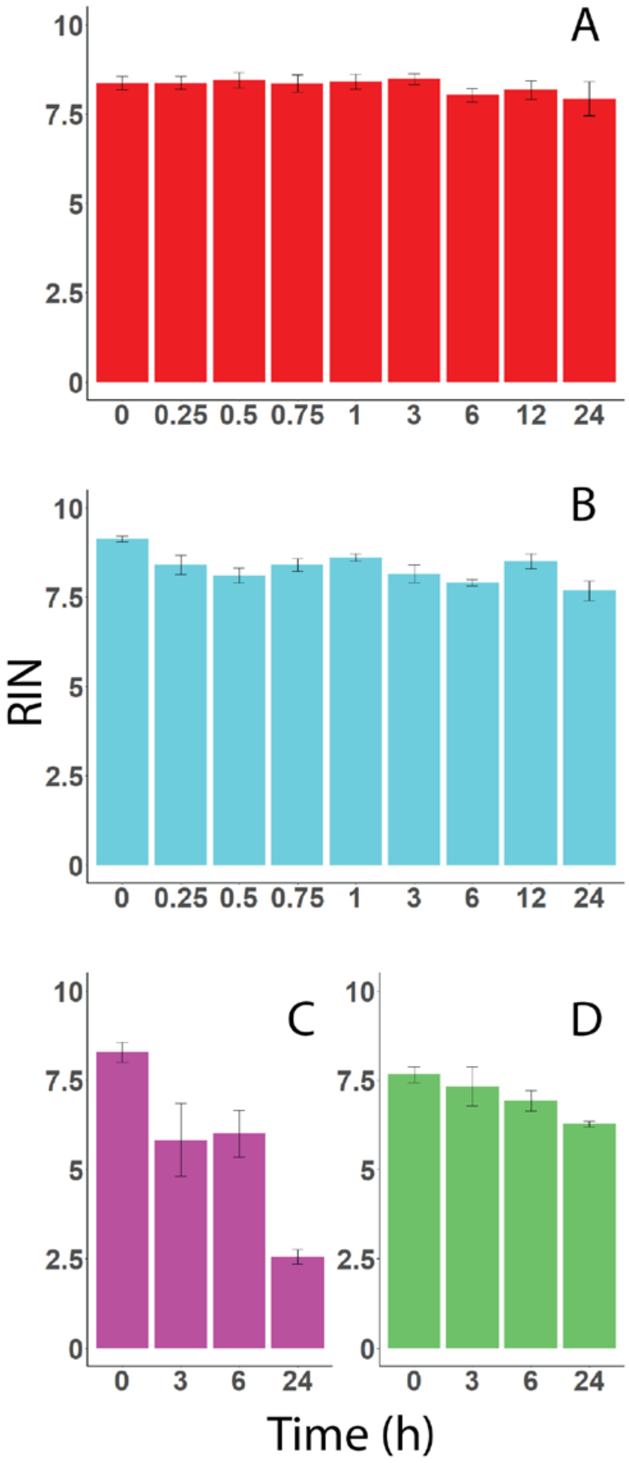

Figure S2. RNA integrety numbers (RINs). A). Mouse retina B). Mouse RPE/choroid. C). Baboon retina. D). Baboon RPE/choroid.

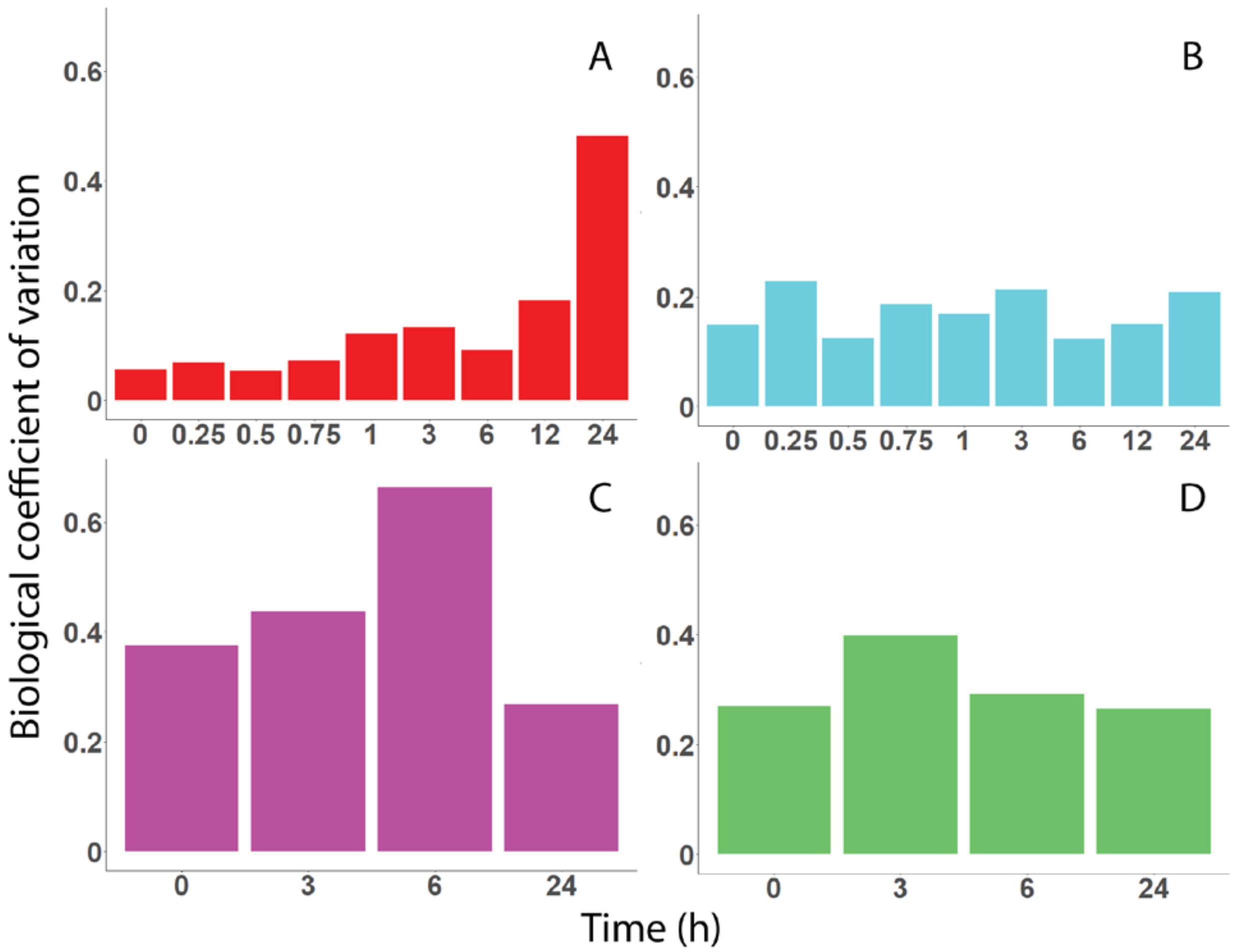

Figure S3. Biological coefficient of variation. A). Mouse retina B). Baboon retina C). Mouse RPE/choroid tissues. D). Baboon RPE/choroid tissues.

mouse

A

PMI (h)	0	0.25	0.5	0.75	1	3	6	12	24
0		2170	2226	2524	2531	2630	4455	5897	2199
0.25	619		0	6	191	2100	3236	4778	1793
0.5	3741	0		0	150	1890	2940	4454	1672
0.75	373	0	0		0	940	2370	4468	1627
1	1606	0	1727	0		108	1518	3847	1141
3	2694	86	1532	0	0		82	2990	738
6	7413	4330	6655	3814	2265	119		881	540
12	9450	8241	9122	7948	6006	5170	3477		0
24	9619	9409	10110	9201	7926	7609	7263	2572	

Retina

RPE/choroid

baboon

B

PMI (h)	0	3	6	24
0		0	4969	9063
3	8715		0	7686
6	2262	3130		5058
24	8571	2407	2309	

Retina

RPE/choroid

Figure S4. Number of differentially expressed genes between all tested PMIs. A). Mouse retina and RPE/choroid. B). Baboon retina and RPE/choroid.

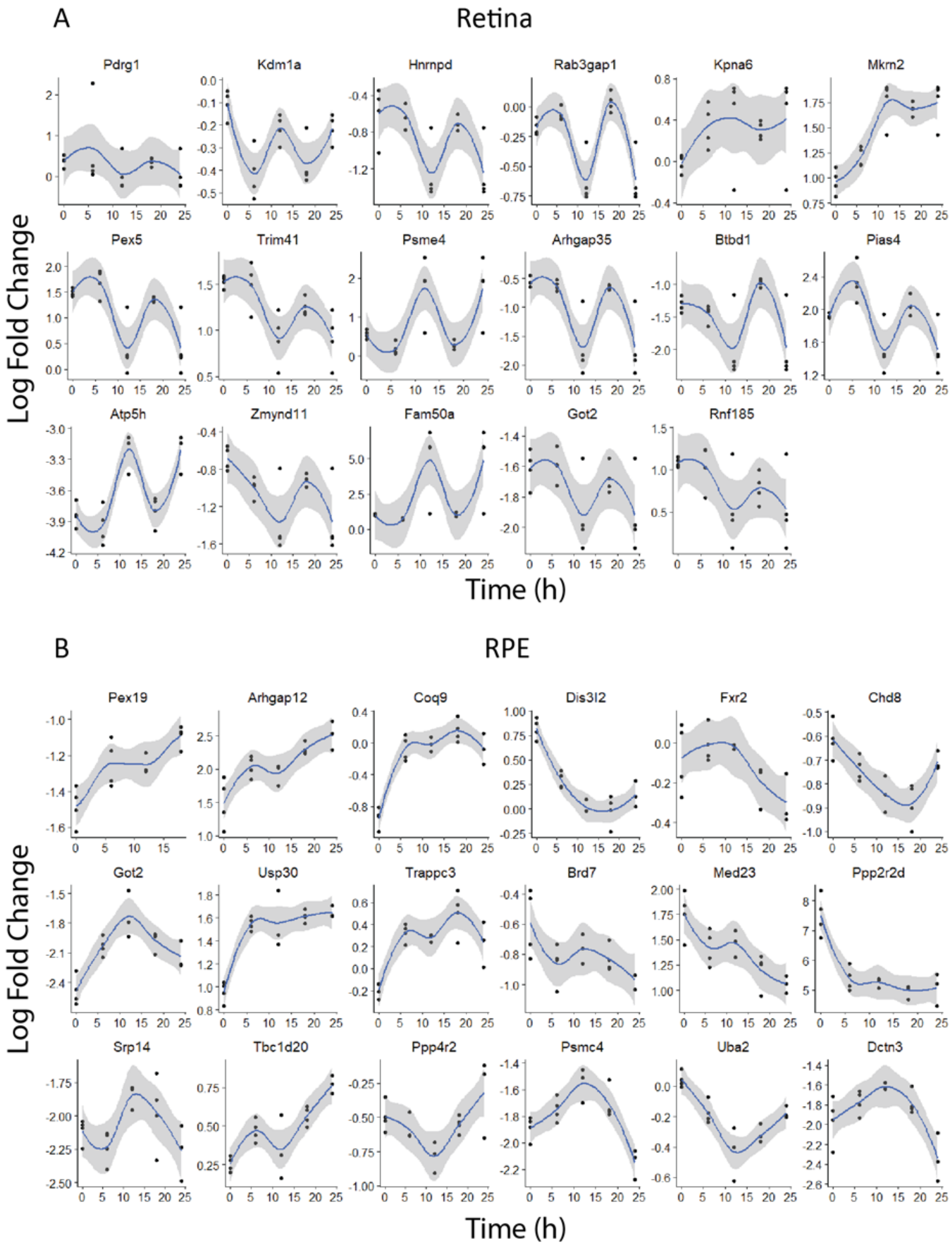

Figure S5. qRT-PCR results of selected mouse genes used for PMI modeling. A). Retina B). RPE/choroid

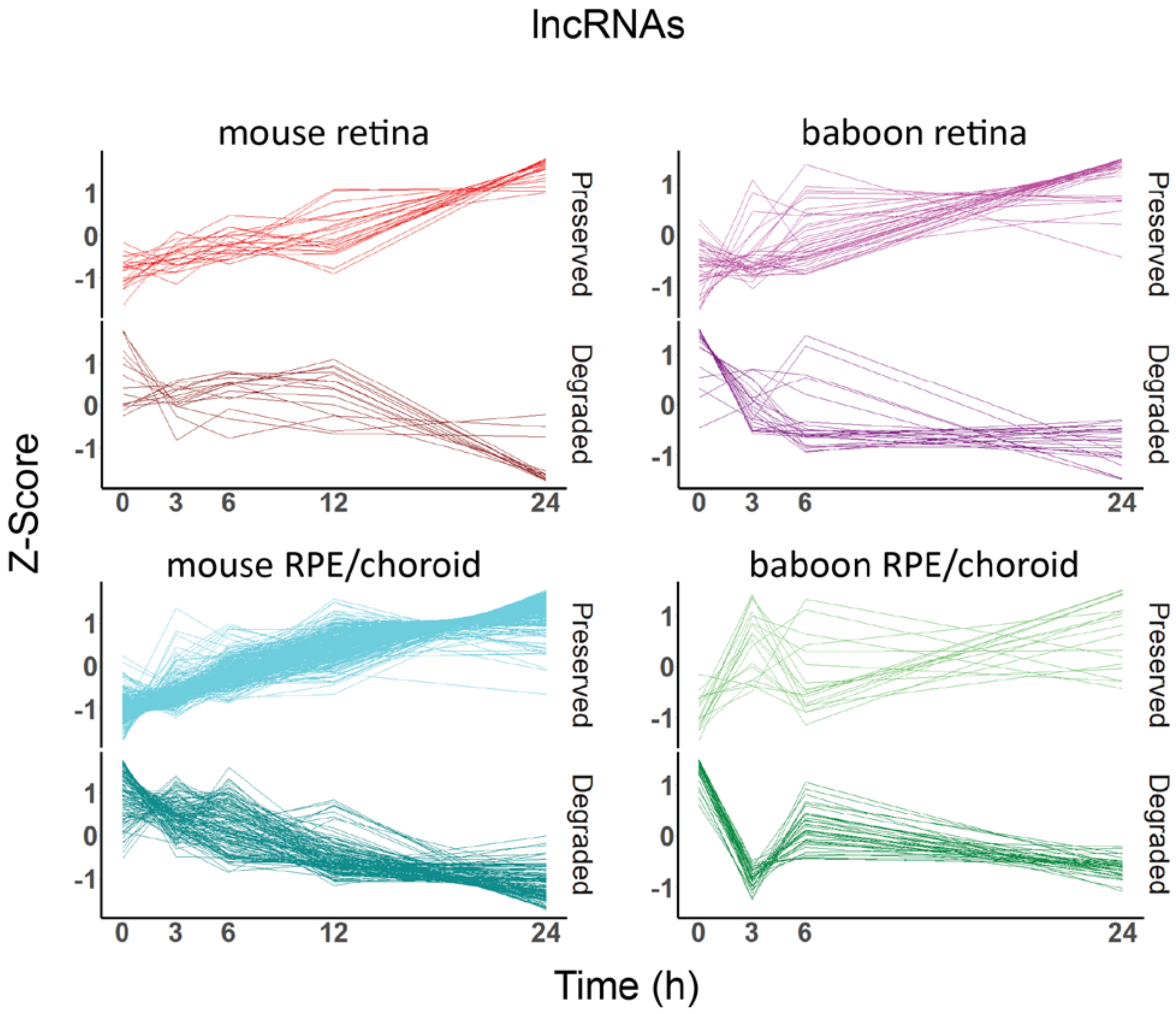

Figure S6. Z-Scores of preserved and degraded differentially expressed lncRNAs.

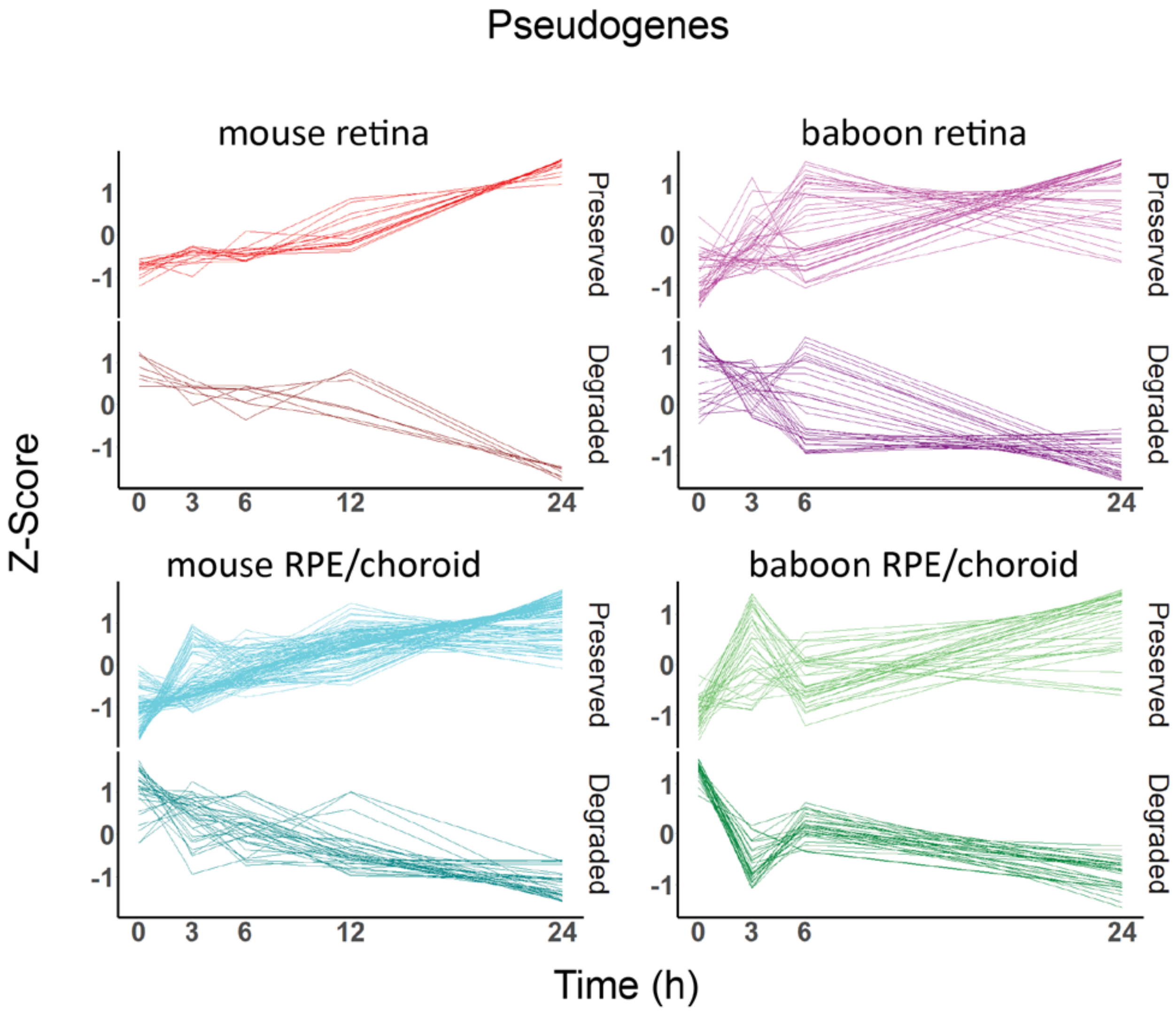

Figure S7. Z-Scores of preserved and degraded differentially expressed pseudogenes.

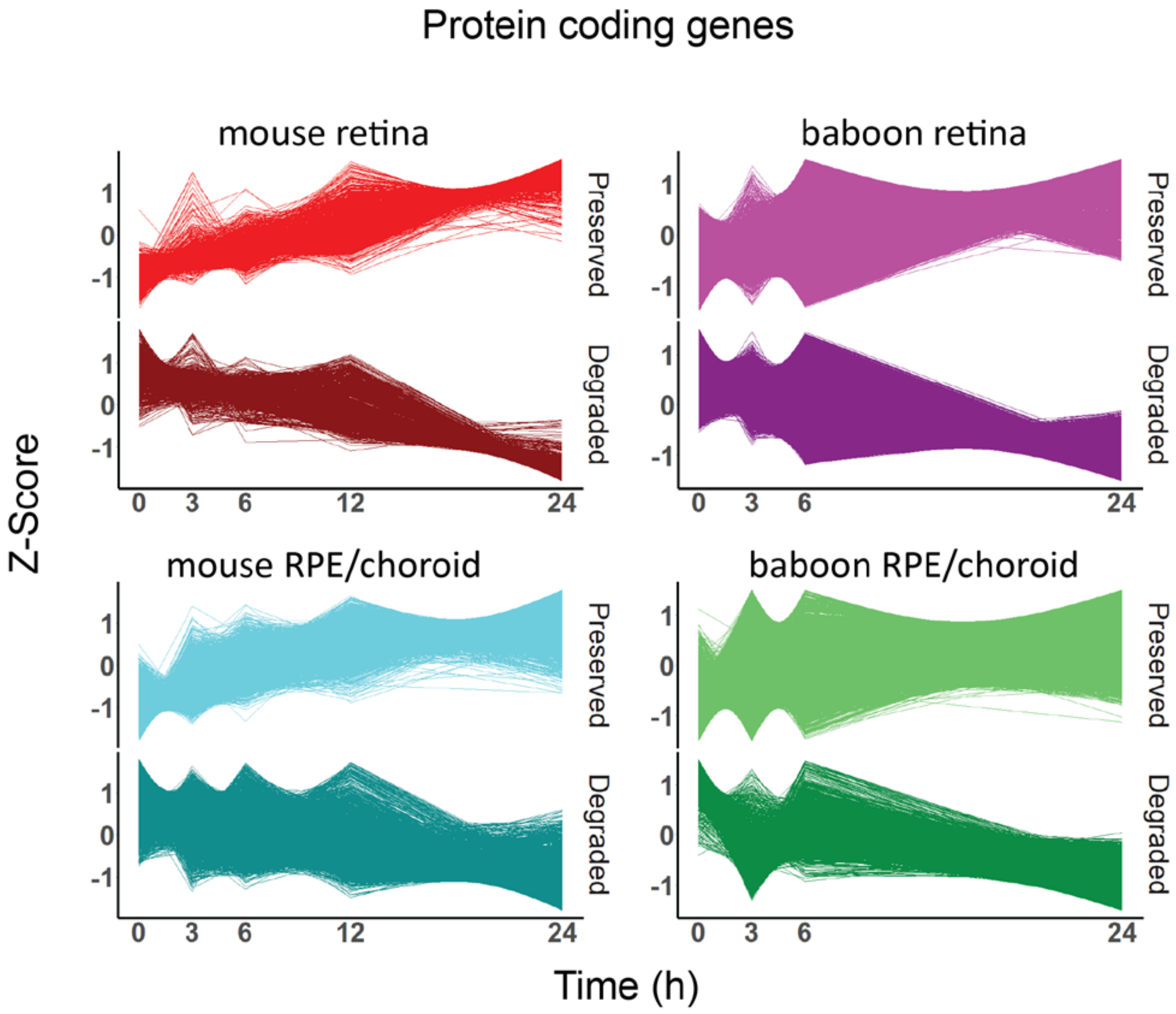

Figure S8. Z-Scores of preserved and degraded differentially expressed protein coding genes.
